# The Effect of Mobile Care Delivery on Clinically Meaningful Outcomes, Satisfaction, and Engagement Among Physical Therapy Patients: Observational Retrospective Study

**DOI:** 10.2196/31349

**Published:** 2022-02-02

**Authors:** Lauren Beresford, Todd Norwood

**Affiliations:** 1 Omada Health Inc San Francisco, CA United States

**Keywords:** physical therapy, mobile apps, engagement, health care delivery

## Abstract

**Background:**

Musculoskeletal care is now delivered via mobile apps as a health care benefit. Although preliminary evidence shows that the clinical outcomes of mobile musculoskeletal care are comparable with those of in-person care, no research has examined the features of app-based care that secure these outcomes.

**Objective:**

Drawing on the literature around in-person physical therapy, this study examines how patient-provider relationships and program engagement in app-based physical therapy affect clinically meaningful improvements in pain, function, and patient satisfaction. It then evaluates the effects of patient-provider relationships forged through in-app messages or video visits and timely, direct access to care on patients’ engagement in their recovery.

**Methods:**

We conducted an observational, retrospective study of 814 pre- and postsurveyed participants enrolled in a mobile app physical therapy program where physical therapists prescribed workouts, education, and therapeutic activities after a video evaluation from February 2019 to December 2020. We estimated generalized linear models with logit functions to evaluate the effect of program engagement on clinical outcomes, minimal clinically important differences (MCIDs) in pain (ΔVisual Analogue Scale ≤−1.5) and function (ΔPatient Specific Functional Scale ≥1.3), and the effects of patient-provider relationships and clinical outcomes on patient satisfaction—participant reported likelihood to recommend the program (Net Promoter Scores of 9-10). We estimated Poisson generalized linear models to evaluate the effects of stronger patient-provider relationships and timely access to physical therapy within 24 hours on engagement including the number of weekly workouts and weeks in the program.

**Results:**

The odds that participants (N=814) had a pain MCID increased by 13% (odds ratio [OR] 1.13, 95% CI 1.04-1.23; *P*=.003) with each weekly workout and the odds of a function MCID by 4% (OR 1.04, 95% CI 1.00-1.08; *P*=.03) with each week in the program. Participants with MCIDs in function and large changes in pain (Δ Visual Analogue Scale ≤−3.5) were 1.85 (95% CI 1.17-2.93; *P*=.01) and 2.84 times (95% CI 1.68-4.78; *P*<.001) more satisfied, respectively. Those with video follow-up visits were 2 to 3 times (*P*=.01) more satisfied. Each physical therapist’s message increased weekly workouts by 11% (OR 1.11, 95% CI 1.07-1.16; *P*<.001). Video follow-up visits increased weekly workouts by at least 16% (OR 1.16, 95% CI 1.04-1.29; *P*=.01) and weeks in the program at least 8% (OR 1.08, 95% CI 1.01-1.14; *P*=.02). Access was associated with a 14% increase (OR 1.14, 95% CI 1.05-1.24; *P*=.003) in weekly workouts.

**Conclusions:**

Similar to in-person care, program engagement positively affects clinical outcomes, and strong patient-provider relationships positively affect satisfaction. In app-based physical therapy, clinical outcomes positively affect patient satisfaction. Timely access to care and strong patient-provider relationships, particularly those forged through video visits, affect engagement.

## Introduction

### Background

Physical therapists, providers with highly specialized knowledge in managing musculoskeletal conditions [1-3], now deliver care directly through mobile apps. In a digital environment, physical therapists evaluate and diagnose patients on demand to ensure they receive appropriate care. Rather than prescribing opioids [4-7] or administering unnecessary imaging [8-19], physical therapists can prescribe exercise and education, which are key components of evidence-based care in physical therapy, as a first line of defense [11-13].

Increasing evidence supports that physical therapy via a mobile app delivers pain and functional outcomes comparable with those of in-person care [14-16]. However, this literature does not explore what drives clinically meaningful outcomes in pain, function, and patient satisfaction—the foundational measures of evidence-based physical therapy— in a digital setting [17].

In brick-and-mortar physical therapy clinics, “adherence” to a course of provider-prescribed care drives clinical outcomes [18]. Consistent at-home exercise, which is among the most supported physical therapy interventions, as well as completion of prescribed or insurance-allowed visits are assessed by physical therapists to measure adherence [17,19,20].

Physical therapy delivered through a mobile app may not be structured similarly to in-person physical therapy with a specific number of weekly visits. In the program examined in this paper, care delivery focused on immediate access to care, ad hoc follow-up video visits, and direct, asynchronous communication between patients and their designated therapists. After an initial synchronous video evaluation, physical therapists designed recovery programs to accord with patients’ goals and altered these programs in response to synchronous and asynchronous feedback from patients. Physical therapists guided their patients through phases of their recovery in real time based on their activity levels, feedback to exercises, and changes in pain and function levels throughout an episode of care.

Owing to the real-time nature of physical therapy in this setting, we take a broader view of adherence and measure it as program engagement defined by 2 measures: the number of patient-recorded in-app–prescribed therapeutic weekly workouts and the number of weeks participants are active in the program. We first tested the hypothesis that clinical outcomes (clinically meaningful pain reduction and functional improvement) were positively associated with program engagement.

In concert with driving clinical outcomes by leveraging the best available evidence, evidence-based care is patient-centered, which is measured by patient satisfaction [21]. Some evidence indicates that patient satisfaction with in-person physical therapy is based on office experiences such as wait times and friendly exchanges between patients, physical therapists, and office staff [22-24], whereas digital care removes such experiences. However, care delivered through an app can nurture relationships between physical therapists and patients through in-app chat and face-to-face video visits. We tested the hypothesis that the strength of patient-provider relationships [25,26] measured by the frequency of digital communication with providers (number of days providers send weekly in-app chat messages and number of synchronous follow-up video visits) is positively associated with patient satisfaction.

There is inconsistent evidence in the literature about how patients’ clinical outcomes affect patient satisfaction with physical therapy [27]. By removing some of the subjective aspects of care (eg, appointment wait times, office cleanliness, friendliness of staff), clinical outcomes may take on new significance for patient satisfaction in a digital setting. Therefore, we also tested the hypothesis that patient satisfaction is associated with the clinical outcomes of the program itself.

There are explicit trade-offs between care delivered through a mobile app versus in-person office visits. On the one hand, regular face-to-face visits may better strengthen patient–provider relationships than app-based video visits and chats. On the other hand, patients who arrive at in-person physical therapy only after referral, ineffective self-management, or alternative therapies (eg, acupuncture and massage), may be less motivated to engage in their treatment than those who can directly access care the same day via an app. Although we cannot interrogate these trade-offs in this paper, our secondary purpose is to understand if the strength of digital patient-provider relationships and immediate access to care via a mobile physical therapy program affects how readily participants engage in their own recovery.

In traditional clinical settings, provider communication with patients affects their adherence to treatment [28], which, in turn, affects whether patients experience meaningful clinical outcomes. Interpersonal connections with providers often motivate patients to adhere to prescribed care [24,29]. Patients’ relationships with their providers are strengthened the more they interact [25,26]. The content of communication also matters; positive feedback from providers is associated with exercise adherence [20]. The providers in this study were trained to positively reinforce exercise adherence via in-app chat and video visits. We hypothesize that the frequency of patient-provider digital communication is associated with physical therapy program engagement as measured by longer episodes of care and more weekly workouts.

There is also evidence that early, direct access to physical therapy can affect clinical outcomes by treating conditions before they become more chronic and difficult to treat [30-32]. This effect may be behavioral in the sense that patients who are motivated and able to expediently address an issue are more likely to engage and do the hard work to get better, that is, to exercise [33]. By reducing barriers to access physical therapy, patients, regardless of their chronicity, who are motivated to initiate physical therapy can promptly do so, and this motivation may express itself in better engagement than those who wait longer for initial video evaluations [34]. We tested the hypothesis that access to initial evaluations with physical therapists within 24 hours is associated with greater program engagement.

### Objective

Our goal in this study is to examine the aspects of patient-provider relationships and program engagement that are associated with clinically important differences in pain and function along with patient satisfaction in physical therapy delivered via a mobile app. The secondary purpose of this study is to understand how 2 aspects of mobile app–based care delivery—relationships built on in-app interactions and immediate access to care—affect patient behaviors that are clinically meaningful: consistently working out and sticking with the program.

## Methods

### Study Design

We conducted an observational, longitudinal, retrospective study using data collected from commercial users of a physical therapy program delivered via a mobile app offered as a health benefit with no cost or copay to privately insured employees by their employers [35]. The study used health care operations data, not originally collected for research purposes, which were deidentified for analysis. Participants registered and checked for program eligibility through a landing page created specifically for their employer and accessible through employers’ benefits portals. Once eligibility was verified, participants were given a passphrase to download the app, read and accept in-app informed consent, and complete a mandatory in-app baseline survey. Each survey response was associated with an individual participant’s account. The Western Institutional Review Board granted an exemption from human subject research for the study’s protocol.

We used established patient-reported outcome measures, including the Patient Specific Functional Scale (PSFS), Visual Analogue Scale (VAS), and Global Rate of Change (GROC), which were delivered asynchronously [36]. Our internal user experience team developed the layout and functionality of the surveys. Both the baseline and final surveys surfaced questions in the same order for all participants. Before launching the program on February 15, 2019, we deployed the surveys to other populations of patients treated in the program. The survey results from this trial period demonstrated that they consistently agreed with the patients’ subjective reports.

The baseline survey had an average of 4 questions across 5 screens. All the questions in the baseline survey were required to be answered. The final survey had an average of 4.5 questions across 4 screens, with responses to all but one open-ended question required. Participants could go back during their surveys and edit responses on previous pages, but they could not review their responses as a summary or alter their surveys after submission.

### Intervention

To enroll in the program, participants created an account in a mobile app and entered demographic information (age and gender), their chief complaint, and provided pain and function ratings in an in-app baseline survey. The participants were matched with a therapist licensed in their state to schedule an initial video evaluation visit. The program’s therapists were trained in evidence-based approaches to evaluate, diagnose, and treat patients on demand via a mobile app.

During the evaluation, physical therapists conducted an in-depth interview and performed a physical exam over secure in-app video to establish a functional baseline and arrive at a diagnosis. On the basis of the participant’s diagnosis and treatment goals, physical therapists then prescribed a course of care accessible through the app. Therapists also assigned educational content specific to patients’ conditions, therapeutic activities (eg, icing or going for a walk), and asynchronous digital physical assessments. Physical therapists modified their patients’ care plans in response to direct feedback from patients via in-app chat, regular pain and function surveys, or follow-up video visits.

All activities in the program were collected and quantified, including completion of prescribed in-app exercises and therapeutic activities, in-app chats with physical therapists, and subsequent video visits. At the end of the program, participants were asked to complete a final survey, which included final measures of pain and function.

### Participants

We included participants in the study who enrolled after the launch of the program on February 15, 2019, and completed the program by December 31, 2020, if they were (1) aged ≥18 years; and (2) presented with a musculoskeletal condition such as low back pain, neck pain, arthritis, sprains, strains, or similar overuse injuries that would benefit from physical therapy or presented for postoperative rehabilitation; and (3) completed a participant survey of clinical outcomes at the end of their episode of care or reported reliable pain and function metrics toward the end of care in weekly surveys. We excluded participants if they (1) did not meet the inclusion criteria and (2) endorsed symptoms or multiple conditions during the initial video evaluation that physical therapists determined would preclude the use of app-based physical therapy as a first line of treatment and required referral for an in-person physical exam (eg, fractures, cervical central cord lesion, subarachnoid hemorrhage or ischemic stroke, unexplained weight gain or loss, fatigue and malaise, among other conditions).

Participants in our sample were not automatically excluded if they endorsed symptoms found on the Optimal Screening for Prediction of Referral and Outcome-Review of Systems (OSPRO-ROS) tool [37]. Rather, physical therapists assessed the appropriateness of app-based physical therapy given patients’ explanations of their symptoms and the ongoing management of those conditions by a physician.

During the study period, 945 participants completed the program and a final outcome survey. Participants typically completed the voluntary final survey within 2 weeks of finishing the program and were neither incentivized nor reminded to do so. We carried forward 33 pain and function observations that participants reported in weekly in-app pain and function surveys if participants reported them less than 3 weeks before completing the program and more than 2 weeks after starting the program. Weekly pain and function surveys were not implemented until September 23, 2020, and participants responded more readily to these earlier in their recovery, resulting in few responses to carry forward. We also imputed 32 values for missing satisfaction scores using the modal responses of similar participants with similar earlier in-episode satisfaction scores. The average time between baseline and outcome responses collected during either the final survey or last weekly pain and function surveys was approximately 44 days.

To eliminate outliers, we calculated the standardized individual difference by dividing participant-level pre–post outcome differences by the SD of those differences and eliminating observations above and below 1% of the distribution for both clinical outcomes [38]. If participants reported differences in pain or functional scores outside of these thresholds, they were excluded. This procedure eliminated 128 outliers. Most of these outliers (all but 29) had inconsistent data where participants reported improvement on the GROC final survey question (GROC>0) but reported that either their pain or function worsened and were moving in opposing directions.

**Figure 1:**
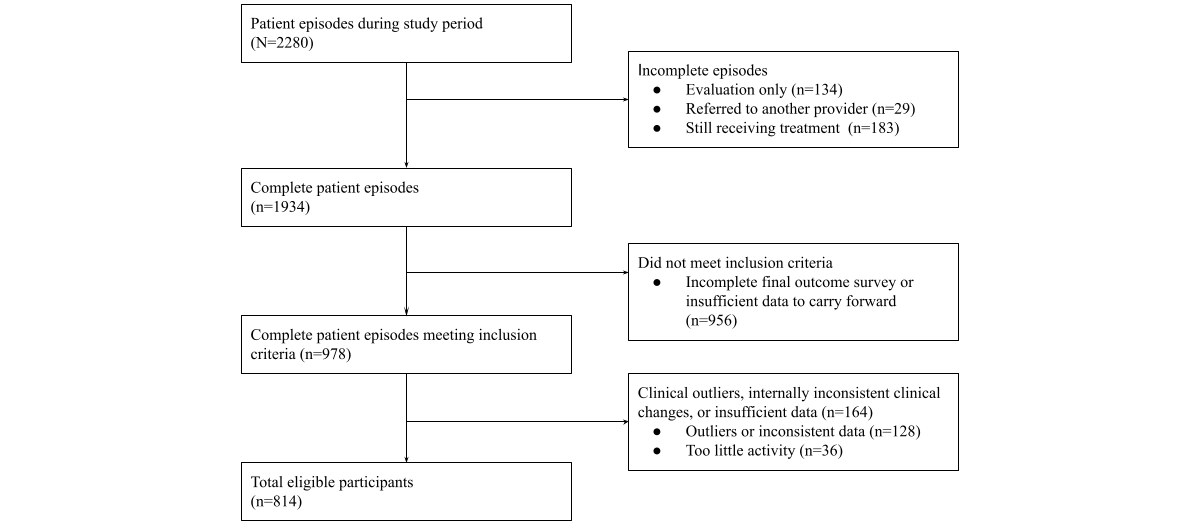
Study participation flow diagram.

A total of 36 participants had too little activity to make reliable conclusions about the program’s outcomes (no workouts and <2 weeks in the program) and were excluded from the analysis. This left a total of 814 eligible participants included in the study (Figure 1). We estimated models with and without outlier removal, with 2.5% outlier elimination, as well as with and without carrying forward the final pain and function observations and obtained similar results.

### Measurements

#### Clinical Outcomes

In the baseline and end-of-program surveys, participants rated their maximum pain levels over the last 24 hours using the VAS [39] on a scale from 0 (“no pain”) to 10 (“worst pain imaginable”). Participants rated their level of functional impairment on a scale from 0 (“completely unable to perform”) to 10 (“able to perform normally”) for up to 3 different self-identified activities impacted by their condition using the PSFS [36,39,40]. We used the functional measure for the activity participants mentioned first because this is likely the daily activity that they struggle with most. We also modeled the average score across the PSFS activities, which yielded similar results, but resulted in a greater number of outliers. In the app, participants saw these scales as a slider that ranged continuously from 0 to 10.

We created 2 binary variables for minimal clinically important differences (MCID) in pain (VAS) and function (PSFS): a value of 1 was assigned to participants’ episodes with changes in their pain ≤−1.5 points and ≥1.3 points in their functional ability [36]. Otherwise, a value of 0 was assigned.

We also created binary variables equal to 1 for large changes in pain (ΔVAS≤−3.5) and function (ΔPSFS≥2.7) and 0 otherwise based on thresholds identified in the literature [36]. We did this because, at the outset of the study, we did not know if different thresholds of change in clinical outcomes might affect satisfaction because clinical outcomes are not observed to affect satisfaction in the literature evaluating in-person physical therapy. We found that thresholds for moderate changes in pain (−3<ΔVAS≤−3) or function (2.7<ΔPSFS≥2.3) contained relatively few observations (61 observations for those with moderate pain changes and 36 for those with moderate function changes) and did not affect satisfaction; therefore, we decided to test the effects of MCIDs on pain and function and large changes in these clinical outcomes, retaining the smallest change necessary to affect satisfaction [36].

#### Satisfaction

Satisfaction with the program was measured by a final survey question that was used to calculate the Net Promoter Score (NPS) by asking participants to answer: “How likely is it that you will recommend the program to a friend or colleague?” on a scale from 0 “Not at all Likely” to 10 “Extremely Likely.” NPS defines categories of respondents as “Detractors” (0-6), “Passives” (7-8), and “Promoters” (9-10) [41,42]. Owing to the lack of variation in this variable, we chose to investigate the correlates of being a promoter. We created a binary variable equal to 1 if participants scored the NPS question with a score of 9 or 10 and 0 otherwise.

#### Program Engagement

Two variables measured program engagement: (1) the number of in-app workouts per week and (2) the duration of the program in weeks. The duration of the program was calculated as the difference between the time participants started the program after their initial evaluation to the end of the program, which was defined as the time when patients were either discharged directly by their provider or were inactive for 2 weeks, whichever came first.

#### Patient-Provider Relationships

During the program, physical therapists communicated asynchronously with participants through in-app chat to assess their progress and provide guidance. Physical therapists and participants also scheduled synchronous follow-up video visits. Patient-provider communications are used to measure the strength of these relationships and are captured by (1) the number of unique days a provider sends a message to participants through in-app chat per week and (2) a categorical variable for the number of video follow-up visits after participants’ initial video evaluations. The categories for follow-up visits included (1) no visits, (2) 1 to 2 visits, (3) 3 to 4 visits, and (4) 5 or more visits. The category for “no visits” was omitted from our models to serve as a comparator.

#### Access

Prompt access to care was measured in days to the initial video evaluation after enrolling in the program. A binary variable was created with 1 assigned to those who accessed care within 24 hours, and 0 assigned to those who accessed care after 24 hours.

#### Controls

Chronicity, baseline pain and function levels, comorbid conditions, and adverse symptoms can affect participants’ recovery [14]. We controlled for comorbid conditions including hypertension, diabetes, cardiovascular disease, a family or personal history of cancer, or other conditions, including behavioral health conditions. We also controlled for adverse symptoms found on the OSPRO-ROS [37], such as night sweats, headaches, lightheadedness, or abnormal sensations.

We controlled for baseline pain and function. Baseline pain was categorized as little to no pain (VAS≤1), mild pain (3.4≤VAS>1), moderate pain (7.4≤VAS>3.4), and severe pain (VAS>7.4) based on cut points identified in the literature [43]. Severe baseline pain (other categories were omitted) as well as continuous baseline pain and function scores served as controls in our models because, in our clinical practice, we observed that patients with poorer scores on baseline pain and function face larger physical and behavioral health obstacles to recovery than patients with better scores, who also have less room to improve [44,45]. We present controls in the results when they are statistically significant (*P*<.05).

### Statistical Analysis Plan

To test our hypotheses, we estimated generalized linear models (GLMs). GLMs for MCIDs in pain, function, and satisfaction were estimated using the binomial family of exponential dispersion models and a logit link function, which is equivalent to a logistic regression model fit by maximum likelihood estimation. GLMs for the number of workouts per week and number of weeks in the program were estimated using the Poisson family of exponential dispersion models and a log link function. We interpreted our results by evaluating changes in the odds of an outcome, which were calculated by exponentiating the coefficients from the model, and by subtracting 1 from the odds to better interpret odds that were less than one (negative coefficients).

## Results

### Overview

Table 1 presents the demographic and clinical profiles of the participants in the sample at baseline. Nearly half (387/814, 47.5%) of the participants were female and aged approximately 41 years, on average. Furthermore, 26.2% (214/814) were aged ≥50 years when musculoskeletal symptoms present with greater frequency, limiting productivity while working [46]. The participants were treated for various musculoskeletal conditions. No single anatomical region captured most of the participants’ diagnosed conditions.

Table 2 presents descriptive statistics for outcomes and predictors in the analysis. Mean VAS was 1.7 (SD 1.9) at program completion compared with 4.4 (SD 2.2) at baseline (Table 1). Approximately 66.8% (544/814) experienced an MCID in pain (VAS Δ≤–1.5) with 35.5% (289/814) experiencing a large pain change (VAS Δ≤–3.5) [36]. Mean PSFS was 7.8 (SD 2.4) post treatment, compared with 5.2 (SD 3) at baseline (Table 1), with nearly 63.7% (519/814) reporting an MCID in function (PSFS Δ≥1.3) and 51.7% (421/814) a large change (PSFS Δ≥2.7) [36]. Participants were highly satisfied with an average 9.3 on the NPS question. The average participant logged 2.8 workouts per week over an average duration of 9.1 weeks in the program.

On average, providers frequently communicated with the participants. About one-third (257/814, 31.6%) of the participants completed 3 or more additional video visits beyond the initial evaluation. In between visits, physical therapists checked in with participants about 1.8 days per week via in-app chat. Provider chat messages consisted of single messages or in-depth live chat conversations with participants. Approximately 52.8% (430/814) of the participants completed their initial video consultation within 24 hours of registering for the program. Multimedia Appendix 1 provides a heatmap of significant Pearson correlations between the variables included in the analysis.

**Table 1:** Baseline participant characteristics (N=814).

Characteristics				Values
**Demographics**				
	Female, n (%)		387 (47.5)	
	**Age (years)**			
		Value, mean (SD)	40.85 (11.9)	
		≥50, n (%)	214 (26.3)	
**Anatomical region, n (%)**				
	Low back pain		172 (21.1)	
	Shoulder		132 (16.2)	
	Knee		118 (14.5)	
	Neck		104 (12.8)	
	Upper body, elbow, wrist, hand, or arm		84 (10.2)	
	Lower body, ankle, foot or leg		83 (10.3)	
	Hip		70 (8.6)	
	Back or spine		46 (5.7)	
	Other		5 (0.6)	
**Clinical baseline, mean (SD)**				
	Pain baseline (VAS^a^)		4.4 (2.2)	
	Function baseline (PSFS^b^)		5.2 (3.0)	
**Baseline pain level categories, n (%)**				
	Little to no pain (VAS≤1)		61 (7.5)	
	Mild pain (3.4≤VAS>1)		218 (26.8)	
	Moderate (7.4≤VAS>3.4)		475 (58.4)	
	Severe pain (VAS>7.4)		60 (7.4)	
**Chronicity, n (%)**				
	Chronic (>3 months)		497 (61.1)	
	Subacute (1-3 months)		128 (15.7)	
	Acute (<1 month)		189 (23.2)	
**Comorbid conditions and adverse symptoms**, **n (%)**				
	Reported comorbid conditions		383 (47.1)	
	Reported adverse symptoms		281 (34.5)	

^a^VAS: Visual Analogue Scale.

^b^PSFS: Patient Specific Functional Scale.

**Table 2:** Descriptive statistics for outcomes and predictors (N=814).

Variables				Values
**Clinical outcomes**				
	Pain outcome (VAS^a^), mean (SD)			1.7 (1.9)
	**Pain changes, n (%)**			
		Pain MCID^b^ (ΔVAS≤−1.5)	544 (66.8)	
		Large pain MCID (ΔVAS≤−3.5)	289 (35.5)	
	Function Outcome (PSFS^c^), mean (SD)			7.8 (2.365)
	**Function changes, n (%)**			
		Function MCID (ΔPSFS≥1.3)	519 (63.8)	
		Large function MCID (ΔPSFS≥2.7)	421 (51.7)	
**Satisfaction**				
	Likelihood to recommend, mean (SD)			9.3 (1.5)
	Promoters, n (%)			674 (82.8)
**Program engagement, mean (SD)**				
	Number of workouts per week			2.8 (2.2)
	Weeks in program			9.1 (5.4)
**Patient-provider communication**				
	Days messaged by physical therapist per week, mean (SD)			1.8 (1.1)
	**Follow-up visits, n (%)**			
		None	232 (28.5)	
		1-2	325 (39.9)	
		3-4	180 (22.1)	
		≥5	77 (9.5)	
**Access, n (%)**				
	24 hours to first visit			430 (52.8)

^a^VAS: Visual Analogue Scale.

^b^MCID: minimal clinically important difference.

^c^PSFS: Patient Specific Functional Scale.

### Clinical Outcomes

**Figure 2:**
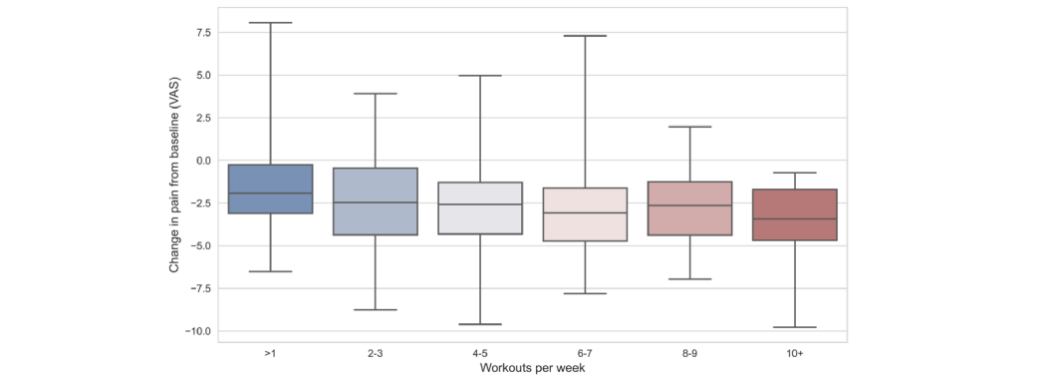
Distribution of pain change by number of workouts. VAS: Visual Analogue Scale.

Figure 2 demonstrates that as weekly workouts increased, pain decreased. In Table 3, we see that after controlling for significant baseline characteristics, the odds of having an MCID in pain increased by 1.13 (*P*=.003) times for each additional weekly workout a participant completed. There were no significant direct effects of access or the strength of patient-provider relationships as proxied by patient-provider communication on MCIDs in pain or function.

Participants’ baseline chronicity and pain affected the odds of having an MCID in pain. We observed a 46% (*P*<.001) reduction in the odds of having an MCID in pain among participants with chronic conditions compared to those with conditions that troubled them for less than 3 months. Those with severe pain saw a 70% (*P*=.01) reduction in the odds of having an MCID in pain compared to those with less severe pain levels. However, the odds of having an MCID in pain increased by 80% (*P*<.001) for each additional unit in reported baseline pain; those with higher pain, all else being equal, had more room to improve their pain.

**Figure 3:**
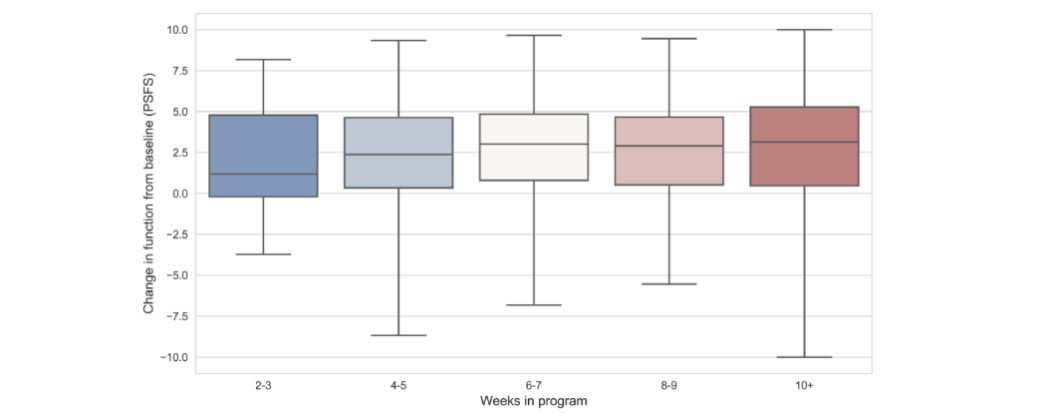
Distribution of functional change by weeks in program. PSFS: Patient Specific Functional Scale.

Figure 3 illustrates the positive relationship between functional improvements and weeks in the program. Table 3 further shows that program engagement also increased the odds of having an MCID in function, but only as measured by weeks in the program and not the number of workouts per week. We observed a 4% (*P*=.03) increase in the odds of having an MCID in function with each additional week a participant spent in the program.

Participants’ age, chronicity, and baseline pain severity and function affected the odds that the participants saw an MCID in function. The odds of completing the program with an MCID in function were 53% (*P*<.001) lower for participants aged ≥50 years than those of younger participants. Similar to the results for pain, the odds of having an MCID in function were 50% (*P*<.001) lower for participants with chronic conditions compared to their counterparts with acute and subacute conditions. The odds of having an MCID in function were also 71% (*P*<.001) lower for participants with severe pain compared to those with moderate, mild, or little to no pain. With each additional unit of reported baseline function, the odds of having an MCID in function decreased by 42% (*P*<.001); better functioning patients had less room for improvement.

**Table 3:** Odds ratios (ORs) for generalized linear models of program engagement and baseline controls on clinical outcomes (814 observations)^a^.

Variables					OR (95% CI)			*P* value
**Pain MCID^b^**								
	Intercept			0.13 (0.07-0.24)			<.001	
	**Program engagement**							
		Number of workouts per week	1.13 (1.04-1.23)			.003		
	**Controls**							
		Age ≥50 years	0.56 (0.38-0.83)			.003		
		Chronic condition	0.54 (0.38-0.77)			<.001		
		Severe pain	0.30 (0.12-0.74)			.01		
		Baseline pain	1.80 (1.63-2.00)			<.001		
**Function MCID**								
	Intercept			89.24 (43.52-182.98)			<.001	
	**Program engagement**							
		Number of weeks in program	1.04 (1.00-1.08)			.03		
	**Controls**							
		Age ≥50 years	0.47 (0.31-0.72)			<.001		
		Chronic condition	0.50 (0.35-0.74)			<.001		
		Severe pain	0.29 (0.14-0.57)			<.001		
		Baseline function	0.58 (0.54-0.63)			<.001		

^a^Comorbid conditions, adverse symptoms, and access were not significant, and there was no direct relationship between provider communication and outcomes.

^b^MCID: minimal clinically important difference.

### Satisfaction

Table 4 presents the results of our GLM for satisfaction, which was measured using a binary variable for whether a participant was a “promoter” of the program. Satisfaction was positively related to video follow-up visits by providers (increasing odds of being a promoter 2 to 3 times). The odds of being a promoter were 85% (*P*<.001) higher if participants had an MCID in function. However, improvements in pain only significantly affected the odds of being a promoter if participants experienced large pain changes. Participants with large changes in pain had nearly 3 times the odds (odds ratio 2.84, 95% CI 1.68-4.78; *P*<.001) of being a promoter of the program compared to those with smaller or no pain changes.

**Table 4:** Odds ratios (ORs) for generalized linear models of strength of patient–provider relationships, clinical outcomes, and baseline controls on satisfaction (814 observations)^a^.

Variables					OR (95% CI)			*P* value
**Promoter (likelihood to recommend ≥9)**								
	Intercept			1.47 (0.56-3.84)			.43	
	**Patient**–**provider communication**							
		1-2 follow-up visits	2.06 (1.33-3.20)			<.001		
		3-4 follow-up visits	2.17 (1.27-3.70)			.01		
		≥5 follow-up visits	3.32 (1.42-7.79)			.01		
	**Pain and function changes**							
		Function MCID^b^	1.85 (1.17-2.93)			.01		
		Large pain MCID	2.84 (1.68-4.78)			<.001		
	**Controls**							
		Female	2.23 (1.48-3.34)			<.001		
		Baseline function	1.09 (1.01-1.17)			.03		
		Baseline pain	0.85 (0.22-0.95)			.004		

^a^A total of 32 imputed values (782 original).

^b^MCID: minimal clinically important difference.

### Program Engagement

#### Patient-Provider Relationships

**Figure 4:**
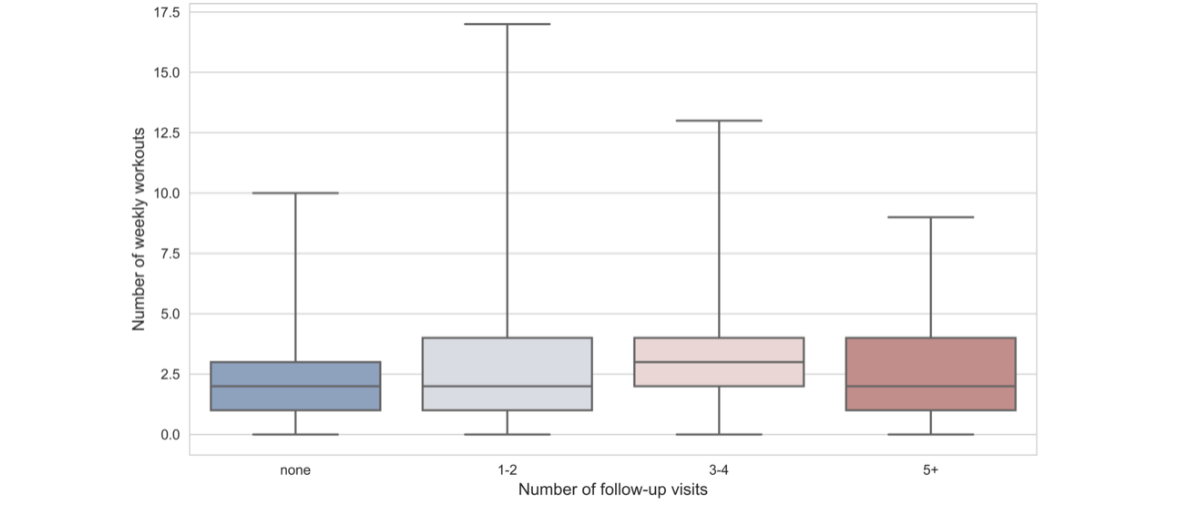
Distribution of number of workouts per week by number of follow-up visits.

Table 5 presents results for program engagement measured by number of workouts per week and weeks in the program. Each additional weekly message a physical therapist sent to participants increased the number of workouts per week by 11% (*P*<.001). Follow-up visits also directly affected the number of weekly workouts that participants completed. In Figure 4, we demonstrate the relationship between the frequency of video follow-up visits and number of weekly workouts.

The results in Table 5 show that, compared with participants who did not have follow-up visits, those with 1 to 2 follow-up video visits had 16% (*P*=.01) more workouts per week and those with 3 to 4 follow-up visits had 32% (*P*<.001) more workouts per week. This effect tapered off and was no longer significant for participants with 5 or more follow-up visits. These results indicate that there may be a sweet spot for on-screen facetime between patients and providers to build a strong, motivating relationship.

**Table 5:** Odds ratios (ORs) for generalized linear models of strength of patient–provider relationships, access, and baseline controls on program engagement (814 observations).

Variables				OR (95% CI)		*P* value
**Number of workouts per week**						
	Intercept		2.04 (1.68-2.47)		<.001	
	**Patient-provider communication**					
		1-2 follow-up visits	1.16 (1.04-1.29)		.01	
		3-4 follow-up visits	1.32 (1.17-1.49)		<.001	
		≥5 follow-up visits	1.06 (0.90 to 1.25)		.49	
		Days messaged by physical therapist per week	1.11 (1.07-1.16)		<.001	
	**Access**					
		24 h to first visit	1.14 (1.05-1.24)		.003	
	**Controls**					
		Age ≥50 years	1.25 (1.14-1.37)		<.001	
		Adverse symptoms	0.87 (0.79-0.95)		.002	
		Severe pain (VAS^a^>7.4)	0.76 (0.63-0.92)		.01	
		Baseline pain (VAS)	1.02 (1.00-1.05)		.049	
		Baseline function (PSFS^b^)	0.96 (0.95-0.98)		<.001	
**Number of weeks in program**						
	Intercept		9.57 (8.80-10.40)		<.001	
	**Patient-provider communication**					
		1-2 follow-up visits	1.08 (1.01-1.14)		.02	
		3-4 follow-up visits	1.28 (1.19-1.36)		<.001	
		≥5 follow-up visits	1.91 (1.77-2.05)		<.001	
		Days messaged by physical therapist per week	0.85 (0.83-0.87)		<.001	
	**Controls**					
		Age ≥50 years	1.11 (1.05-1.16)		<.001	
		Chronic Condition (>3 months)	1.13 (1.08-1.18)		<.001	
		Adverse symptoms	1.11 (1.06-1.17)		<.001	
		Baseline function (PSFS)	0.99 (0.98-0.99)		<.001	

^a^VAS: Visual Analogue Scale.

^b^PSFS: Patient Specific Functional Scale.

#### Access

Table 5 also shows that participants had greater odds of completing more workouts per week if they accessed physical therapy quickly through an initial video evaluation within 24 hours. Participants who saw their physical therapist within 24 hours finished 14% (*P*=.003) more weekly workouts than those who waited longer for visits.

We included significant controls for age, adverse symptoms, pain severity, and baseline pain and function scores. Interestingly, participants aged ≥50 years had about 25% (*P*<.001) more weekly workouts than their younger counterparts. These participants may have had more time to work out (eg, fewer small children at home) or they may have been more motivated to work out to ease persistent conditions.

Participants who concurrently experienced adverse symptoms found on the OSPRO-ROS did approximately 13% (*P*=.002) fewer weekly workouts compared with those who did not present with these symptoms. Those with severe pain also had fewer weekly workouts, despite an inverse relationship between worse baseline pain and function scores and program engagement via working out.

Table 5 additionally shows the effect of patient–provider relationships on the number of weeks participants remained in the program. Although program duration is not the ideal measurement of engagement, it further validates our findings on how patient–provider communication may strengthen relationships and its association with program engagement and meaningful functional outcomes.

**Figure 5:**
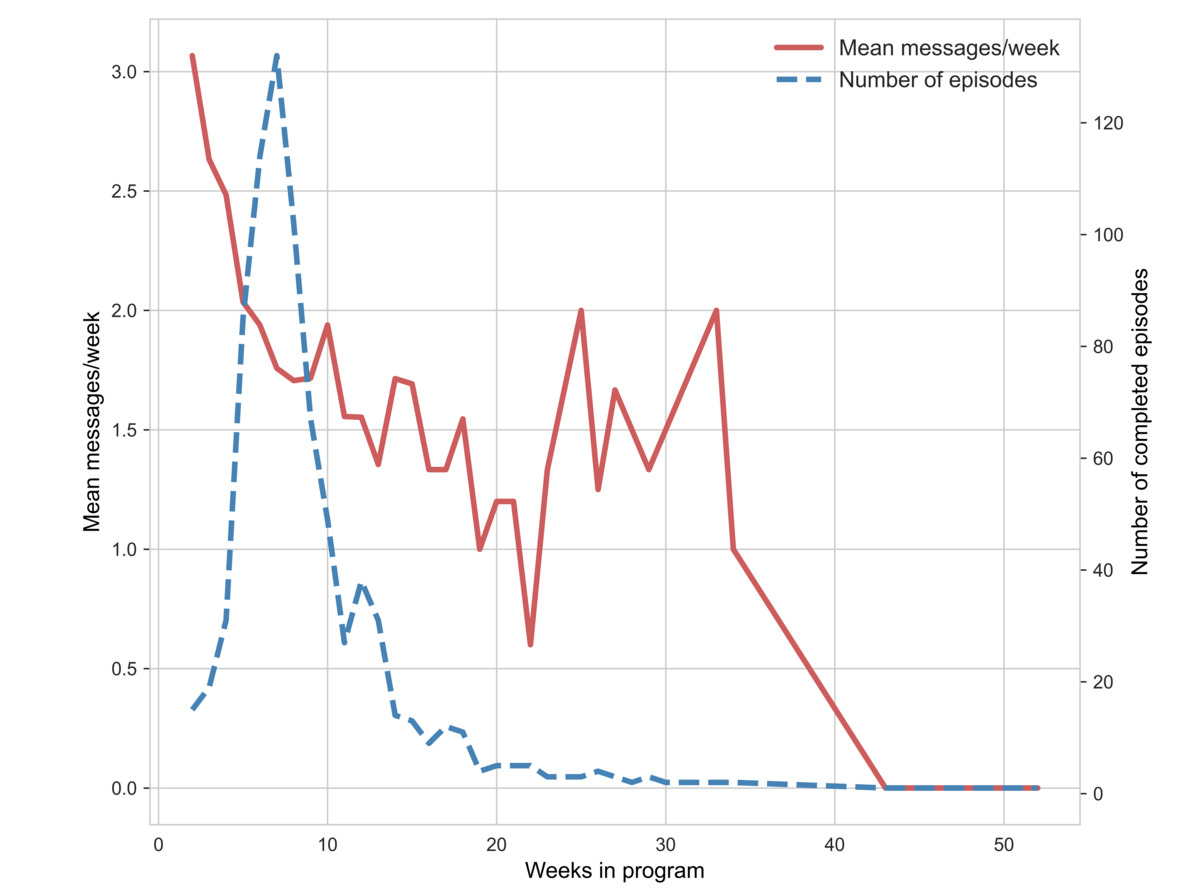
Mean physical therapist messages per week by distribution of program durations in weeks.

Table 5 shows a negative association between the number of weekly physical therapists’ messages and weeks in the program. Each additional weekly message sent by a physical therapist to the participants was associated with a 15% decrease in the number of weeks in the program (*P*<.001). As depicted in Figure 5, this may be because of unsuccessful attempts to reach out to participants who achieved their program goals, but had not communicated with their physical therapists who, therefore, delayed formal discharge.

Additional video follow-up visits were positively associated with program duration. Compared with participants who did not have follow-up visits, Table 5 shows that those with 1 to 2 follow-up video visits had 8% (*P*=.02) more weeks in their episodes, those with 3 to 4 follow-up visits had 28% (*P*<.001) more weeks, and those with ≥5 follow-up visits spent 91% (*P*<.001) more weeks in the program. Program access within 24 hours was not significantly correlated with the program duration.

Participants who were aged ≥50 years (*P*<.001) with chronic conditions (*P*<.001) and adverse symptoms (*P*<.001) all had greater odds of having longer episodes than their younger counterparts without chronic conditions or adverse symptoms. Those with higher functionality at baseline had shorter episodes (*P*<.001). Multimedia Appendix 2 illustrates all models with the main effects only.

## Discussion

### Principal Findings

This study builds on prior studies that show that mobile app–based physical therapy delivers similar outcomes to in-person care [16,47,48]. Similar to in-person physical therapy, clinical outcomes for physical therapy delivered via a mobile app were positively associated with program engagement [17-20]. Meaningful changes in pain were positively correlated with participants performing the most clinically relevant activity: consistently exercising.

However, the mechanism driving clinically meaningful changes in function requires a different form of engagement: time in the program. Exercise-induced analgesia is well documented in the literature, although the mechanism remains unclear [49,50]. We speculate that patients may associate exercise with pain reduction because they perceive a change in tissue status after stretching and movement. They may report they feel “looser” or “more flexible” immediately after exercise and associate that as a positive result. Changes in function may occur gradually, with incremental improvements not perceived until they hit a specific functional threshold, resulting in a change in task performance, such as more easily picking up their child or walking down the stairs. As changes in function are likely grounded in changes in strength, range of motion, motor planning, or motor control, several weeks of consistent exercise may be required to achieve meaningful functional improvement. Future research should explore the causal mechanisms underlying functional improvement versus pain reduction.

Unlike traditional physical therapy, we observed that clinical outcomes were more closely associated with satisfaction. A minimal amount of functional change had a large effect on participants’ willingness to recommend the program under study. However, it took large changes in pain to influence participants to recommend the program to their friends and family.

Perceptions of functional changes may differ from perceptions of pain change. In our clinical practice, most patients during intake come to us “because they don’t want to hurt anymore” and expect pain to be eliminated. Patients may have more relative, vague expectations around functional recovery unless they cannot perform activities required for their livelihood. Patients often struggle to pinpoint goals for functional improvement. Pain alone may not be enough for patients to stop doing something altogether or they may not have a requisite daily task that they can no longer perform (eg, they must be able to lift 50 pounds for their job; they cannot pick up their child). This means that the elimination of pain (a hard outcome to achieve) must be met to be satisfied, but a lower level of functional improvement may yield satisfaction. Future research should unpack perceptions around changes in pain and function throughout recovery.

Physical therapy delivered via a mobile app resembles in-person physical therapy in that it depends on strong relationships between patients and providers to be successful. Frequent, albeit not weekly, video follow-up visits were positively associated with satisfaction, the completion of more weekly workouts, and persistence in the program, which were the key ingredients for recovery. Asynchronous messaging may also help strengthen patient–provider relationships because weekly workouts increased with each day per week that providers messaged participants. However, provider messaging may have a negative effect if used to chase unresponsive participants later in the program. Provider messages also did not have a significant effect on satisfaction, whereas video visits did.

Frequent, face-to-face interactions between providers and participants may keep participants motivated and remain active in the program until they see significant improvements. Future research should further explore how digital communication can build stronger therapeutic alliances between physical therapists and patients in a digital setting [22,29].

Unlike traditional in-person physical therapy, mobile physical therapy has the potential to reduce time to care [51,52], with significant effects on program engagement (number of weekly workouts). Patients who seek care can access it immediately, which may have a motivating effect to help them initiate behavioral changes that alleviate pain and restore functionality [34,53]. Direct access removes a barrier to traditional physical therapy, which is often delayed while patients traverse a costly referral process or receive inappropriate care from other providers who do not practice evidence-based care [54]. The experience of being passed from one provider to another is time consuming, frustrating, and may negatively impact patients’ motivation toward recovery. Given the evidence that mobile apps can provide prompt access to care that yields results comparable with in-person care, apps may also deliver better and more cost-effective results than the typical care pathway that begins with a physician [1-3,47,54]. Care delivered via mobile apps also removes barriers to recovery that can make initiating traditional physical therapy inconvenient, including appointment scheduling and travel [55].

We cannot eliminate the possibility that participants who access care sooner are more intrinsically motivated or have fewer barriers to exercising than those who delay their appointments. The delivery of care in a digital environment is a promising area for future research to understand how providers can optimize care to ensure better clinical outcomes and patient satisfaction.

### Limitations

We did not find any direct relationship between clinical outcomes and access to care or patient-provider communication that indicates strong ties. Rather, access and relationships between physical therapists and patients that were strengthened by digital communication were associated with patient behaviors that were then followed by significant recovery outcomes. Future work should aim to understand the causal relationships between the design of mobile app physical therapy programs in terms of access, indicators of different qualities of patient-provider relationships, and the recovery behavior of participants.

This study is inherently limited as an observational study of an employer-based population. The voluntary nature of, and lack of compensation for, completing the final survey meant that our sample size was reduced, potentially biasing our results. The results may not be generalizable to a broader population of employees, retirees, or children. Our study also lacked a control group. Future research should compare meaningful clinical outcomes, satisfaction, and program engagement of mobile app–based physical therapy to in-person physical therapy in a controlled clinical trial. Randomized control trials or other suitable experimental methods should be used to unpack causality around patient-provider communication and relational indicators, access to care, and program engagement.

### Conclusions

Physical therapy delivered via a mobile app may be more likely to result in clinically important changes in pain and function if it engages patients by directly connecting them with physical therapists and by facilitating strong relationships with their providers. Synchronous communication, in particular video visits, may help physical therapists foster strong relationships that personalize app-based care and build in accountability and encouragement so that patients engage in recovery and, concomitantly, enjoy clinically important improvements in pain and function. In app-based physical therapy, clinical outcomes may be more closely associated with patient satisfaction, independent of patients’ relationships with their providers, than what is observed in studies evaluating in-person physical therapy.

## Appendix

Multimedia Appendix 1 Heatmap of significant Pearson correlations (*P*<.05).

Multimedia Appendix 2 Models with main effects only.

## Abbreviations

GLM: generalized linear model
GROC: Global Rate of Change
MCID: minimal clinically important difference
NPS: Net Promoter Score
OSPRO-ROS: Optimal Screening for Prediction of Referral and Outcome-Review of Systems
PSFS: Patient Specific Functional Scale
VAS: Visual Analogue Scale
