# Supplementary figures and images for: The Effect of Mobile Care Delivery on Clinically Meaningful Outcomes, Satisfaction, and Engagement Among Physical Therapy Patients: Observational Retrospective Study
Source: JMIR Rehabil Assist Technol. 2022 Feb 2;9(1):e31349. doi: 10.2196/31349 (PMC8851343; doi:10.2196/31349)

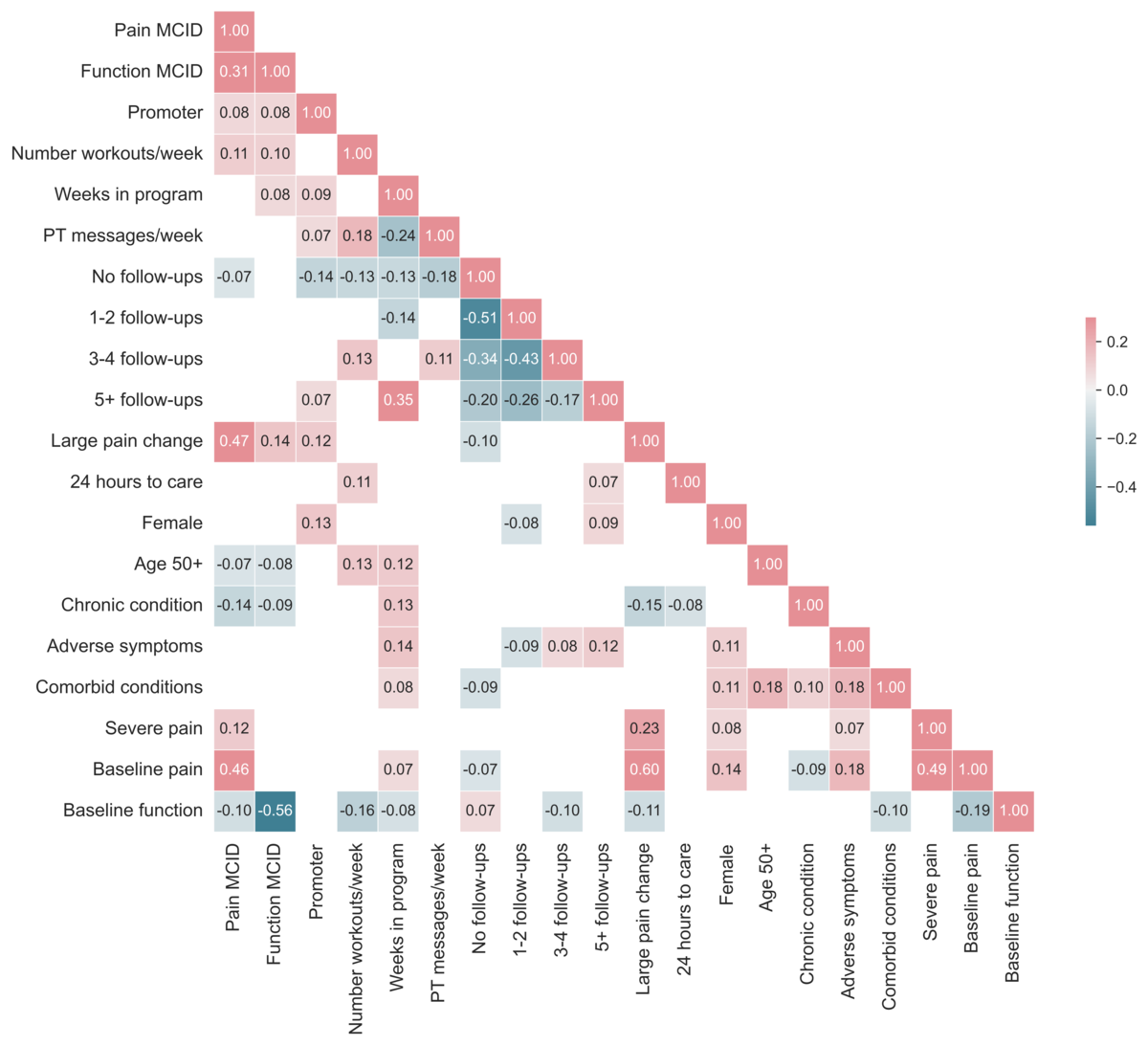

Supplement: Multimedia Appendix 1 [file rehab_v9i1e31349_app1.png]
