# Supplementary material for: The Effect of Mobile Care Delivery on Clinically Meaningful Outcomes, Satisfaction, and Engagement Among Physical Therapy Patients: Observational Retrospective Study
Source: JMIR Rehabil Assist Technol. 2022 Feb 2;9(1):e31349. doi: 10.2196/31349 (PMC8851343; doi:10.2196/31349)
Supplement: Multimedia Appendix 2 [file rehab_v9i1e31349_app2.pdf]

Main Effects Only

Variable			OR (95% CI)	*P* value
**Pain MCID**				
	Intercept		1.47 (1.15 to 1.87)	.002
	Number of Workouts Per Week		1.13 (1.04 to 1.21)	.002
**Function MCID**				
	Intercept		1.32 (0.99 to 1.78)	.06
	Number of Weeks in Program		1.03 (1.00 to 1.06)	.03
**Promoter MCID**				
	Follow-ups Only			
		Intercept	2.87 (2.14 to 3.85)	<.001
		1-2 Follow-up Visits	2.01 (1.32 to 3.08)	<.001
		3-4 Follow-up Visits	2.16 (1.29 to 3.62)	.003
		5+ Follow-up Visits	3.49 (1.52 to 8.01)	.003
	Function MCID Only			
		Intercept	3.68 (279 to 4.86)	<.001
		Function MCID	1.56 (1.08 to 2.25)	.02
	Large Pain Change Only			
		Intercept	3.86 (3.12 to 4.77)	<.001
		Large Pain MCID	2.08 (1.36 to 3.18)	<.001
**Number of Workouts Per Week**				
	Follow-ups Only			
		Intercept	2.32 (2.13 to 2.52)	<.001
		1-2 Follow-up Visits	1.22 (1.1 to 1.36)	<.001
		3-4 Follow-up Visits	1.44 (1.28 to 1.61)	<.001
		5+ Follow-up Visits	1.16 (0.99 to 1.36)	.07
	Days Messaged by PT per Week Only			
		Intercept	2.2 (2.02 to 2.39)	<.001
		Days Messaged by PT per Week	1.13 (1.09 to 1.18)	<.001
	Access Only			
		Intercept	2.54 (2.38 to 2.7)	<.001
		24-hours to first visit	1.18 (1.09 to 1.29)	<.001
**Number of Weeks in Program**				
	Follow-ups Only			
		Intercept	8.00 (7.64 to 8.37)	<.001
		1-2 Follow-up Visits	1.02 (0.96 to 1.08)	.54
		3-4 Follow-up Visits	1.21 (1.13 to 1.29)	<.001
		5+ Follow-up Visits	1.87 (1.74 to 2.01)	<.001
	Days Messaged by PT per Week Only			
		Intercept	11.55 (11.06 to 12.07)	<.001
		Days Messaged by PT per Week	0.87 (0.85 to 0.89)	<.001
